# Aqueous Extract of *Paeonia suffruticosa* Inhibits Migration and Metastasis of Renal Cell Carcinoma Cells via Suppressing VEGFR-3 Pathway

**DOI:** 10.1155/2012/409823

**Published:** 2012-02-08

**Authors:** Shih-Chin Wang, Sai-Wen Tang, Sio-Hong Lam, Chung-Chieh Wang, Yu-Huei Liu, Hsuan-Yuan Lin, Shoei-Sheng Lee, Jung-Yaw Lin

**Affiliations:** ^1^Institute of Biochemistry and Molecular Biology, College of Medicine, National Taiwan University, Taipei 10051, Taiwan; ^2^School of Pharmacy, College of Medicine, National Taiwan University, Taipei 10051, Taiwan; ^3^Department of Pathology, National Taiwan University Hospital, Taipei 10002, Taiwan; ^4^Graduate Institute of Integrated Medicine of Chinese Medicine, China Medical University, Taichung, Taiwan; ^5^Department of Medical Genetics and Medical Research, China Medical University Hospital, Taichung, Taiwan

## Abstract

Renal cell carcinoma (RCC) cells are characterized by strong drug resistance and high metastatic incidence. In this study, the effects of ten kinds of Chinese herbs on RCC cell migration and proliferation were examined. Aqueous extract of *Paeonia suffruticosa* (PS-A) exerted strong inhibitory effects on cancer cell migration, mobility, and invasion. The results of mouse xenograft experiments showed that the treatment of PS-A significantly suppressed tumor growth and pulmonary metastasis. We further found that PS-A markedly decreased expression of VEGF receptor-3 (VEGFR-3) and phosphorylation of FAK in RCC cells. Moreover, the activation of Rac-1, a modulator of cytoskeletal dynamics, was remarkably reduced by PS-A. Additionally, PS-A suppressed polymerization of actin filament as demonstrated by confocal microscopy analysis and decreased the ratio of F-actin to G-actin in RCC cells, suggesting that PS-A inhibits RCC cell migration through modulating VEGFR-3/FAK/Rac-1 pathway to disrupt actin filament polymerization. In conclusion, this research elucidates the effects and molecular mechanism for antimigration of PS-A on RCC cells and suggests PS-A to be a therapeutic or adjuvant strategy for the patients with aggressive RCC.

## 1. Introduction

Chinese herb medicines have been discovered to be potential therapies for various diseases, including diabetes mellitus, tumor, and hypertension [1]. Recently, increasing evidence has shown that some of Chinese herb medicines can be applied to improve the efficiency of conventional cancer therapies and reduce the side effects of chemotherapies for human malignancies, such as breast cancer and prostate cancer [2, 3].

The root cortex of *Paeonia suffruticosa* has been widely used as traditional Chinese herb remedy for treating various diseases, including macula, epilepsy, and menstrual disorders [4]. *Paeonia suffruticosa* was demonstrated to exhibit antiproliferation, anti-inflammation, antiinfection, antihypertention, antidiabetics, and neuroprotective activities [5–8]. The active compounds have been identified from *Paeonia suffruticosa*, such as paeonol and paeoniflorin [9–14]. However, the effect and detailed mechanism for antimigration of *Paeonia suffruticosa* on cancer cells are still unclear.

Renal cell carcinoma (RCC) represents 3% of all human malignancies worldwide with an increasing incidence [15–17]. About 70% of RCCs have been found to be clear cell subtype [18]. The response of RCC cells to traditional therapies, such as the chemotherapy, hormonal therapy, and radiation therapy is very poor [19, 20], and one-fourth of the patients have locally invasive or metastatic RCC [21, 22], resulting in a low five-year survival rate of RCC patients. Several immunotherapies have been used for the treatment of metastatic RCC [23], but only small population of patients with advanced RCC show response with a high toxicity [24].

In this study, we examined the antimigration and antiproliferation effects of the aqueous extracts from ten kinds of Chinese herb medicines, including *Paeonia suffruticosa*, *Trichosanthes kirilowii*, *San jhong kuei jian tang*,* Taraxacum monogolicum*, *Kalimeris indica*, *Platycodi radix*, *Abrus precatorius*, *Catharanthus roseus*, *Andrographis paniculata*, and *Curcuma zedoaria*, and further elucidated the molecular mechanism for the strong antimigration activity of *Paeonia suffruticosa* to RCC cells.

## 2. Materials and Methods

### 2.1. Preparation of Chinese Herb Extracts

**Figure 1:**
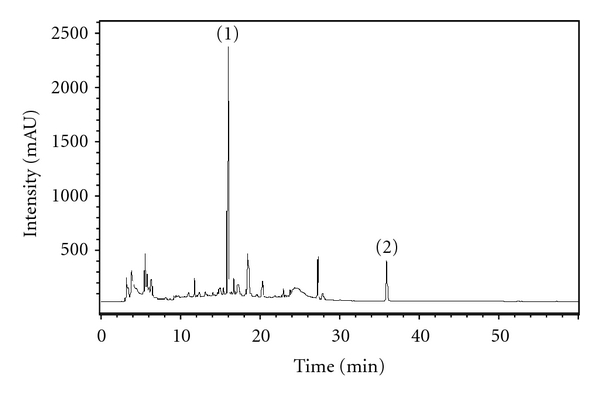
Chemical profile of PS-A by HPLC analysis. Chromatographic patterns of PS-A from HPLC analysis (230 nm) showed peaks corresponding to the retention times (min). Paeoniflorin (1) and paeonol (2) were used as reference compounds for *Paeonia suffruticosa*.

Aqueous extracts from *Paeonia suffruticosa*, *Trichosanthes kirilowii*, *San jhong kuei jian tang*, *Taraxacum monogolicum*, *Kalimeris indica*, *Platycodi Radix*, *Abrus precatorius*, *Catharanthus roseus*, *Andrographis paniculata,* and *Curcuma zedoaria* were provided by Sun-Ten Pharmaceutical Company (Taipei, Taiwan). Briefly, one hundred grams of dried Chinese herbs was boiled with 1,500 mL of H_2_O at 100°C for 30 min and was sieved using a 100-mesh sieve. The extracts were concentrated to 100 mL and were filtered through a 200-mesh sieve. The extracts were dried by speed vacuum concentration and then stored at −20°C until used. Furthermore, the chemical profile of aqueous extracts of *Paeonia suffruticosa* (PS-A) was analyzed by using high-pressure liquid chromatograms (HPLC) and shown in Figure 1. Briefly, HPLC was performed using an Agilent 1100 liquid chromatography (Waldbronn, Germany), consisting of a photodiode array detector. The chromatographic separation of samples (20 *μ*L, 20 mg/mL) was carried out on a Phenomenex Prodigy ODS3 100A column (250 × 4.6 mm, 5 *μ*m), eluted with the mixture of water (A) and acetonitrile (B). The linear gradient program was set as A-B (v/v) from 90 : 10 to 5 : 95 in 60 min with a flow rate of 0.6 mL/min. Absorbance was monitored at 230 nm. Paeoniflorin and paeonol were used as reference compounds for *Paeonia suffruticosa* [10].

### 2.2. Chemicals and Reagents

Paeonol was purchased from WAKO (Japan) and paeoniflorin was from Sigma (St. Louis, MO). The HPLC-grade acetonitrile was obtained from Mallinckrodt (KY, USA), and deionized water was prepared from a Barnstead water purification system (Dubuque, IA, USA). Alexa568-phalloidin and Hoechst 33258 were from Invitrogen Co. Antibodies against VEGF receptor-3, phospho-FAK (Y397), and Rac-1 were obtained from Santa Cruz Biotechnology Inc. Actin, immobilon Western, and secondary antibodies were purchased from Millipore Co. EDTA-free protease inhibitor cocktail was from Roche Diagnostics.

### 2.3. Cell Line and Culture

Renal cell carcinoma cell line 786O was obtained from ATCC and maintained with Dulbecco's Modified Eagle Medium containing 10% fetal bovine serum, 2 mM L-glutamine, 0.1 mM nonessential amino acids, and 10 U/mL penicillin/streptomycin at 37°C in a 5% CO_2_ humidified incubator.

### 2.4. Cell Proliferation Assay

786O cells (7 × 10^2^ cells/well) were seeded into 96-well plates overnight and treated with various concentrations of PS-A for 1–3 days. At the end of treatment, the cells were incubated with 100 *μ*L of fresh medium containing 0.5 mg/mL of 3-[4,5-dimethyl-2-thiazolyl]-2,5-diphenyl-2H-tetrazolium bromide (MTT, Sigma). After incubation for 4 h, 100 *μ*L of 10% SDS/0.1 M HCl was added into well to dissolve the crystal. Absorbance of the reaction products was measured at 590 nm with a reference wavelength 650 nm.

### 2.5. Transwell Migration and Invasion Assay

After serum starvation for 24 h, 786O cells (2.5 × 10^4^ cells) were resuspended in 300 *μ*L of serum-free medium containing various concentrations of PS-A and then seeded into transwell chambers with or without 40 *μ*L of matrigel (1 mg/mL, BD) for invasion or migration assay, respectively. The chambers were put into wells of 24-well plates and incubated with 1 mL of complete medium with 10% FBS at 37°C for 6 h. Cells on the bottom side of the membrane were fixed by 1% formaldehyde/PBS for 10 min and stained with 0.2% crystal violet for 60 min. The migrated or invaded cells were counted using an inverted contrast light microscope under 100x magnification.

### 2.6. Wound-Healing Assay

786O cells were full confluent in 12-well plates at the time of scrapping. Cells were wound with P200 pipette tips and washed with PBS. Cells were then incubated with fresh complete medium containing various concentrations of PS-A. Photographs were taken at the same position of the wound at 0 and 6 h after scrapping.

### 2.7. Mouse Xenograft Model

Four-week-old female NOD-SCID mice were obtained from the National Laboratory Animal Center. Approval for animal experiments was obtained from the Institutional Animal Care and Use Committee of College of Medicine, National Taiwan University. For tumor growth experiments, 786O cells (2 × 10^6^ cells) were inoculated subcutaneously into the flank of mice. Two days after injection, mice were randomly divided into two groups (4 mice per group) and oral-administrated with water or PS-A (0.29 g/kg) five times per week. Tumor size was measured with calipers every five day, and mice were sacrificed after 45 days. Tumor volume was evaluated using the formula: 0.52 × (width)^2^ × length [25]. For tumor metastasis experiments, 786O cells (2 × 10^6^ cells) were intravenously inoculated to the lateral tail vein. Two days after injection, mice were randomly divided into two groups (4 mice per group), and oral-administrated with water or PS-A (0.29 g/kg) five times per week and weighted every five day. Mice were sacrificed after 48 days, and the lungs were excised and fixed by 10% of formaldehyde. The metastatic nodules of lungs were counted for evaluation of approximating tumor content of lungs. 

### 2.8. Western Blot Analysis

After serum starvation for 24 h, 786O cells (10^6^ cells) were treated with or without 0.3 mg/mL of PS for 24 h and then lysed by RIPA buffer (50 mM Tris-HCl pH 7.5, 150 mM NaCl, 5 mM EDTA, 1% Triton X-100, 0.1% SDS, and Roche protease inhibitor cocktail). Fifty *μ*g of protein was resolved on 10% SDS-polyacrylamide gel and transferred to polyvinylidene fluoride membranes (Millipore, Bedford, MA). The membrane was incubated with primary specific antibodies to VEGFR-3, phosphor-FAK, or actin, followed by horseradish peroxidase-conjugated secondary antibodies (Chemicon International, Temecula, CA). Signals were visualized using enhanced chemiluminescence detection reagent from Millipore. Band intensities were obtained using a UVP BioImaging System (UVP Inc., Upland, CA).

### 2.9. Rac-1 Activity Assay

786O cells were starved for 24 h, treated with or without 0.3 mg/mL of PS-A for 12 h, and lysed by lysis buffer (50 mM Tris pH 7.6, 150 mM NaCl, 1% Triton X-100, 0.5 mM MgCl_2_, and Roche protease inhibitor cocktail). 800 *μ*g of total lysate was mixed to glutathione beads coupled with the bacterially produced Rac binding domain of Pak (PBD)-GST fusion protein (a gift from Dr. Zee-Fen Chang) by head-to-head rotation at 4°C overnight. After centrifugation at 14,000 rpm for 2 min, the GST-PBD beads were washed five times with 500 *μ*L of lysis buffer by rotating for 10 min and then boiled with SDS sample buffer (125 mM Tris-HCl, pH 6.8, 4% SDS, 5% *β*-mercaptoethanol, 30% glycerol, and 0.2% bromophenol blue dye) for 10 min to release GTP-bound Rac-1 proteins and then subjected to western blot analysis.

### 2.10. Immunofluorescence Analysis

786O cells were seeded on 22 × 22 mm cover slides and then treated with fresh medium containing various concentrations of PS-A for 12 h. Cells were washed by PBS and fixed using 4% formaldehyde/PBS for 10 min. After being permeabilized by 0.25% Triton X-100/PBS, cells were stained by Alexa568-phalloidin and Hoechst 33258 for 15 min. The cover slides were mounted in 80% glycerol and sealed with nail polish. The images were analyzed with a Leica TCS SP5 Spectral Confocal System.

### 2.11. Preparation of F- and G-Actin Fractions

F-actin was separated from G-actin as described previously with some modifications [26]. Briefly, 786O cells (2 × 10^5^ cells) were treated with 0.3 mg/mL of PS-A for 12 h and then incubated with 200 *μ*L actin stabilizing buffer (1% Triton X-100, 1 *μ*g/mL phalloidin and Roche protease inhibitor cocktail) at room temperature for 5 min. The lysate was collected, and F-actin was separated from G-actin by centrifuging at 100,000 ×g at 37°C for 1 h. The supernatant was G-actin-containing fraction, and the pellet containing F-actin was washed by PBS twice and dissolved by 200 *μ*L of actin dissolving buffer (1% Triton X-100, 2% SDS and protease inhibitor cocktail) on ice for 1 h. The F-actin and G-actin fractions were then analyzed by western blotting. The intensity of band in each lane was determined with a Gel-Pro analyzer (Media Cybernetics, GA, USA).

## 3. Results

### 3.1. Aqueous Extracts of *Paeonia suffruticosa* Suppresses RCC Cell Migration

**Figure 2:**
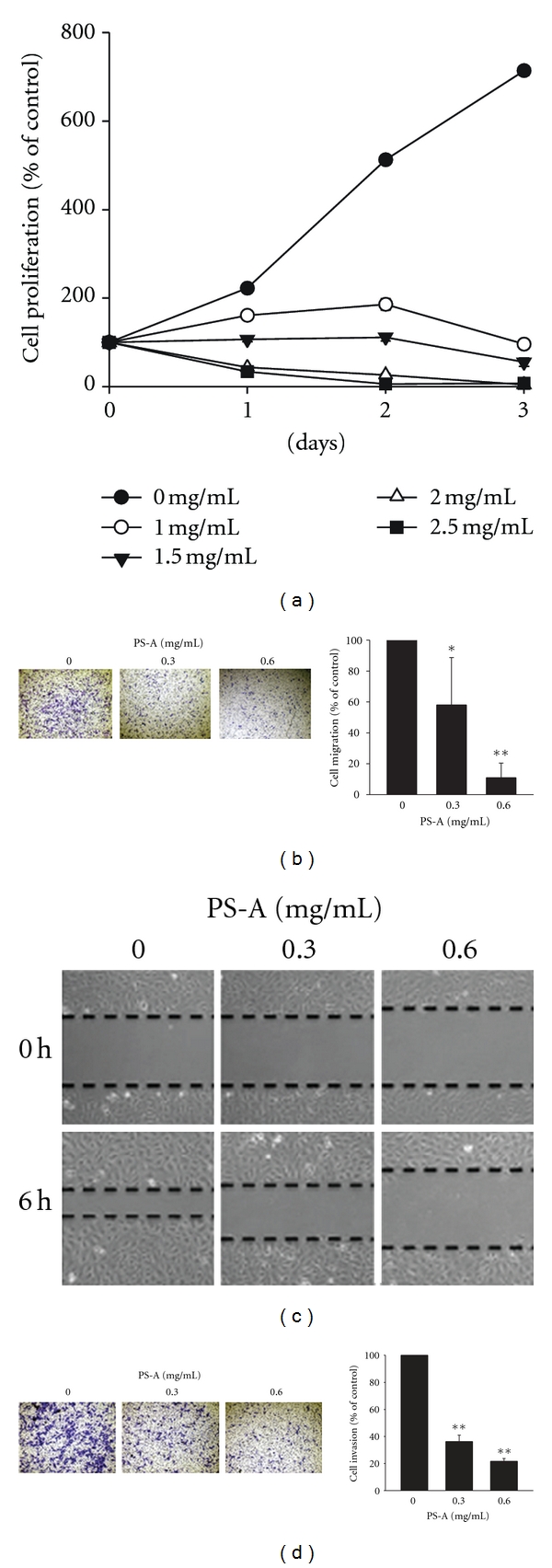
Anticancer effects of PS-A on RCC cells. (a) 7 × 10^2^ of 786O cells were treated with various concentrations of PS-A for 1, 2, and 3 days. The rate of cell proliferation was measured by MTT assay. The data are represented as the means ± SD (*n* = 5). (b) 2.5 × 10^4^ of 786O cells were seeded in transwell chambers and incubated in serum-free medium containing 0, 0.3, and 0.6 mg/mL of PS-A for 12 h. The migrated cells on the bottom of transwell chambers were fixed and then stained. The migratory activity was determined by counting migrated cells in three microscopic fields. The data are represented as the means ± SD (*n* = 3). *P < 0.05; **P < 0.001 compared with untreated cells. (c) Fully confluent 786O cells were scrapped by P200 pipette tips and then treated with 0, 0.3, and 0.6 mg/mL of PS-A. Images were taken at 0 and 6 h after scrapping under 100x magnification. (d) 2.5 × 10^4^ of 786O cells were seeded in matrigel-coated chambers and then incubated in serum-free medium containing 0, 0.3, and 0.6 mg/mL of PS-A for 18 h. The invaded cells were fixed and then stained. The invasive activity was determined by counting invaded cells in three microscopic fields. The data are represented as the means ± SD (*n* = 3). *P < 0.05; **P < 0.001 compared with untreated cells.

To identify active Chinese herb medicines to suppress metastatic process of RCC, aqueous extracts of ten kinds of Chinese herb medicines were prepared for treating 786O cells, the aggressive RCC cell line. The results of MTT assay and transwell migration assay revealed that aqueous extracts of *Paeonia suffruticosa* (PS-A) showed a higher inhibitory effect on cancer cell growth (IC_50  growth_ = 1.5 mg/mL) and a higher ratio between inhibitory effects on cancer cell proliferation and migration in 786O cells (IC_50  growth_/IC_50  migration_ = 5.0), whereas the other nine CHMs showed lower or no inhibitory effects on cell migration activity (Table 1, Figures 2(a) and 2(b)). Additionally, cell mobility and invasion activities of 786O cells were strongly suppressed by the treatment of PS-A (Figures 2(c) and 2(d)). These results suggest that PS-A has strong anticancer activities to RCC cells. 

### 3.2. PS-A Inhibits Tumor Growth and Metastasis *In Vivo*

**Figure 3:**
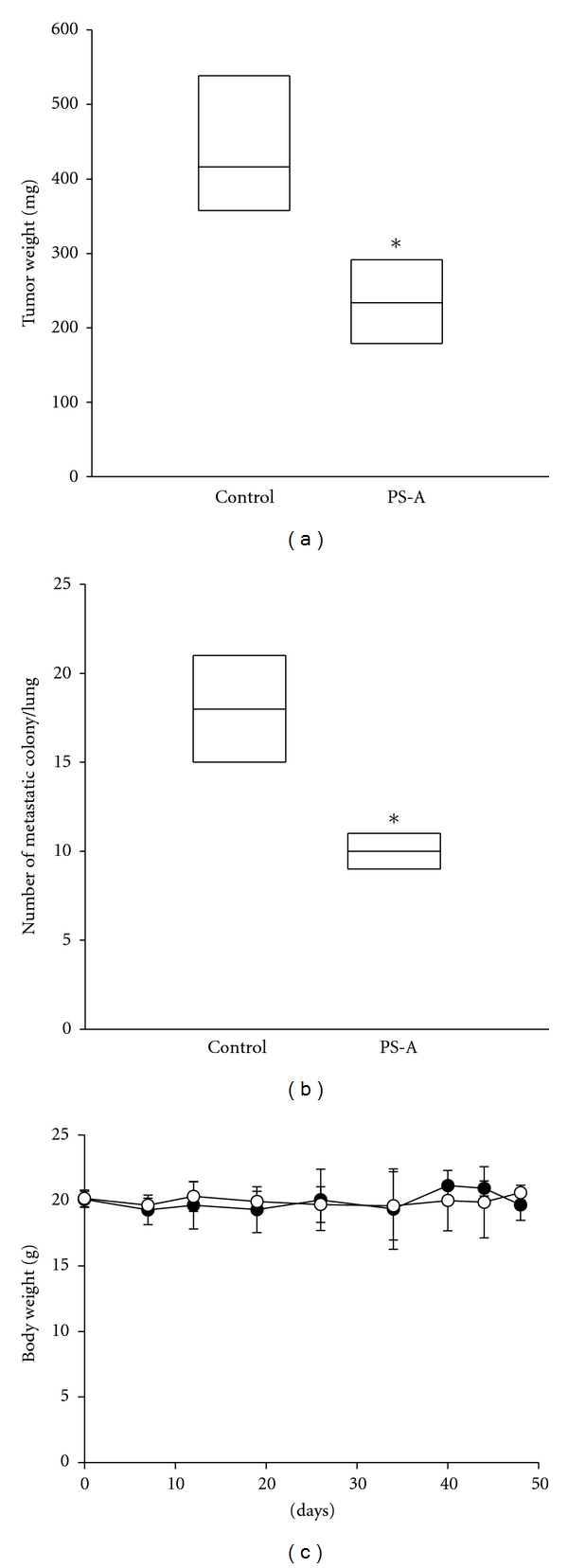
Inhibition of PS-A on tumor growth and metastasis *in vivo*. (a) 2 × 10^6^ of 786O cells were inoculated s.c. into the flank of NOD-SCID mice (*n* = 4). Mice were oral-administrated with water or PS-A (0.29 g/kg) for five days per week. After 45 days, mice were sacrificed and tumors were excised. The weights of tumors are represented as the means ± SD (*n* = 4). *P < 0.05 compared with control. (b) 2 × 10^6^ of 786O cells were inoculated i.v. into the tail vein of NOD-SCID mice (*n* = 4). Mice were oral-administrated with water or PS (0.29 g/kg) for five days per week. After 48 days, mice were sacrificed, and number of metastatic nodules of lung was counted. The number of nodules is represented as the means ± SD (*n* = 4). *P < 0.05 compared with control. (c) Body weight of mice oral-administrated with water or PS-A (0.29 g/kg) for five days per week. Data are represented as the means ± SD (*n* = 4).

To investigate the effects of PS-A on tumor growth* in vivo*, 2 × 10^6^ of 786O cells were injected subcutaneously into the hind limb of NOD-SCID mice, and then the mice were oral-administrated with water or PS-A (0.29 g/kg) five days per week for 45 days. As shown in Figure 3(a), tumor weight of PS-A-treated mice was remarkably lower than that of control group (234.8 mg versus 437.5 mg, resp.; *P* < 0.05). Moreover, 2 × 10^6^ of 786O cells were i.v. injected into lateral tail vein of NOD-SCID mice to examine the effects of PS-A (0.29 g/kg) on the metastatic activity of cancer cells. After 48 days, the number of pulmonary nodules in PS-treated mice was significantly lower than that of untreated group (10 ± 1.2 versus 18.0 ± 3.3 nodules/lung, resp.; *P* < 0.01; Figure 3(b)). Additionally, feeding of PS-A (0.29 g/kg) for 48 days had no significant effect on body weight of mice, suggesting that PS-A showed a very low toxicity to NOD-SCID mice (Figure 3(c)). These results indicate that PS-A inhibits tumor growth and pulmonary metastasis of RCC cells significantly.

### 3.3. PS-A Represses VEGFR-3/FAK/Rac-1 Signaling Pathway

**Figure 4:**
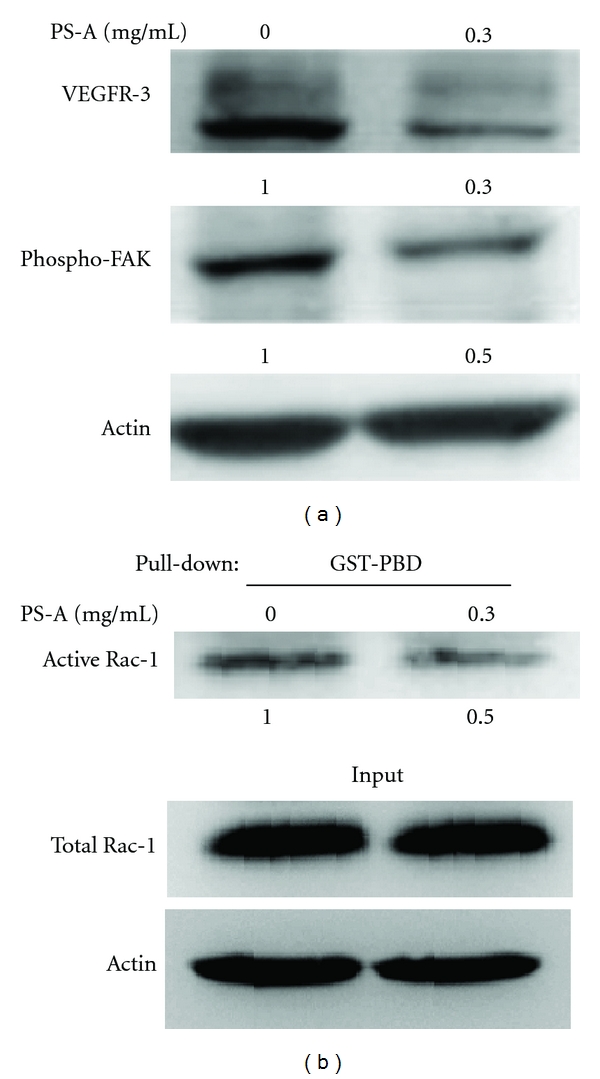
Suppression of VEGFR-3/FAK/Rac-1 signaling by PS-A. (a) After serum starvation for 24 h, 786O cells were treated with or without 0.3 mg/mL of PS-A for 24 h, and the lysates were analyzed by Western blot analysis to detect the expression levels of VEGFR-3 and the phosphorylated status of FAK. Actin was used as the loading control. The intensities of bands were quantitated and normalized to actin levels. (b) After serum starvation for 24 h, 786O cells were treated with or without 0.3 mg/mL of PS-A for 12 h, and the lysates were incubated with GST-PBD beads to examine the levels of GTP-bound Rac-1 (*upper*). The levels of total Rac-1 and actin in total lysates were analyzed by Western blotting (*lower*).

To explore the mechanism for the anticancer activities of PS-A, we examined the effect of PS-A on VEGFRs, which play an important role in tumor cell growth and metastasis [27]. The results revealed that the expression of VEGFR-3 was markedly inhibited by treating 786O cells with PS-A (Figure 4(a)). It has been shown that VEGFRs are involved in the activation of FAK [28]. Therefore, we examined the phosphorylated status of FAK in RCC cells treated with PS-A. The results revealed that the phosphorylation of FAK in PS-A-treated 786O cells was remarkably reduced as compared with that of control cells (Figure 4(a)). Recent reports have shown that phosphorylated FAK is capable of promoting the activation of Rac-1 [29]. To investigate the effects of PS-A on activation of Rac-1, GST-PBD fusion proteins were purified to perform pull-down assay. GTP-bound Rac-1 of 786O cells was significantly decreased by treating with PS-A as demonstrated by pull-down assay, revealing that PS-A reduces the activation of Rac-1 (Figure 4(b)). These results indicate that PS-A suppresses cancer cell migration by inhibiting VEGFR-3/FAK/Rac-1 signaling pathway.

### 3.4. PS-A Reduces the Formation of Actin Filaments of RCC Cells

**Figure 5:**
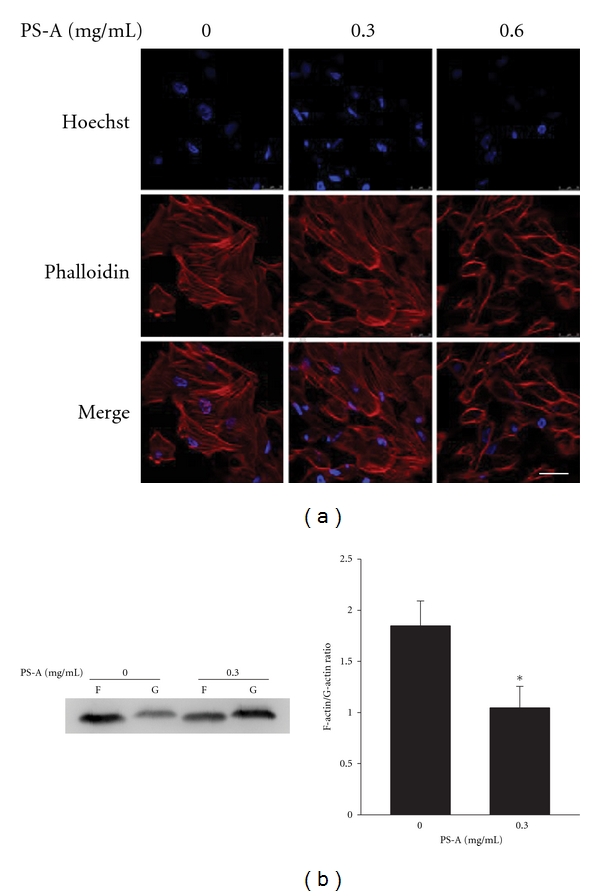
Disruption of actin filament formation by PS-A in RCC cells. (a) 2 × 10^5^ of 786O cells were treated with 0, 0.3, and 0.6 mg/mL of PS-A for 12 h. After fixation, cells were stained with Alexa568-phalloidin (red) and Hoechst 33258 (blue). Fluorescent images were taken with a Leica TCS SP5 Spectral Confocal System. Bars, 20 *μ*m. (b) 2 × 10^5^ of 786O cells were treated with or without 0.3 mg/mL of PS-A, and the fractions containing F- or G-actin were separated and then subjected to Western blotting using actin antibody. The intensities of bands were quantitated, and the ratio of F-actin to G-actin was calculated. The data are represented as the means ± SD (*n* = 3). *P < 0.05 compared with untreated cells.

Recent studies demonstrated that Rac-1 has been reported to be a modulator of cytoskeletal dynamics to affect cell migration [30], and the dynamic change of actin cytoskeleton plays an essential role in cell mobility and migration [31]. Therefore, the effects of PS-A on the formation of actin filaments were studied by staining with phalloidin. The results revealed that PS-A markedly decreased actin filament formation as shown by confocal microscope analysis (Figure 5(a)). The ratio of F-actin to G-actin in PS-A-treated 786O cells was significantly reduced as compared with that of control cells (Figure 5(b)). These results indicate that PS-A disrupts the polymerization of actin filaments to inhibit the migration of RCC cells.

## 4. Discussion

Many studies demonstrated the anticancer activities of Chinese herb medicines in leukemia, colorectal carcinoma, gastric cancer, breast cancer, and prostate cancer [32–36]. Therefore, Chinese herb medicines have been considered as the alternative medicine to treat many tumors which are resistant to traditional cancer therapies. In this study, ten kinds of potential Chinese herb medicines were screened and the aqueous extracts of *Paeonia suffruticosa* exhibited a higher antiproliferation activity to RCC cells. Previous studies showed that *Paeonia suffruticosa* inhibits proliferation in human malignancies including liver cancer [7, 13], esophageal cancer [11, 37], and lung cancer cell lines [14]. Therefore, *Paeonia suffruticosa* could be considered as a potential adjuvant therapy to enhance the efficacy of chemotherapy for patients with aggressive RCC.

VEGFR-3 belongs to the family of receptor tyrosine kinase and is able to activate FAK/Rac-1 pathway [28, 38, 39]. Garces et al. have demonstrated that the NH2-terminus of VEGFR-3 is associated with the COOH terminus of FAK, and disruption of VEGFR3/FAK interaction results in the dissociation of FAK from the focal adhesions [28]. The expression of VEGFR-3 has been found to be upregulated in various human malignancies, including leukemia, breast cancer, colorectal cancer, prostate cancer, renal cell carcinoma, and hepatocellular carcinoma [38, 40]. Expression levels of VEGFR-3 are correlated with portal vein invasion, tumor recurrence, and shorter disease-free survival in HCC patients [41, 42]. It has been shown that the activation of VEGFR-3 participates in cell proliferation, migration, and survival [43]. This study demonstrated that PS-A at the concentration of IC_50  migration_ markedly reduces VEGFR-3 expression and inhibits the activation of VEGFR-3 signaling, indicating that PS-A may have the potential to prevent the progression of human malignancies.

The invasion of cancer cells into adjacent tissues is a critical step in the process of tumor metastasis [44]. Developing of the migratory capacity plays an essential role in cancer cell invasiveness [31]. In this investigation, we demonstrate that PS-A exhibits strong inhibitory effects on the migration, invasion, and metastasis of RCC cells. It might be achieved by disrupting the polymerization of actin filaments, which is tightly regulated during cell migration [45]. Previous studies suggested that the motility of cancer cells is mainly managed by the Rho family of GTPases [46]. Our results showed that the activity of Rac-1, one member of the Rho family, was suppressed by PS-A in RCC cells, indicating the potential mechanism for the inhibitory effects of PS-A to cancer cell migration.

In conclusion, this research demonstrates that PS-A is able to prohibit tumor growth and metastasis through disrupting the formation of actin filament and impairing the signaling pathway of VEGFR-3. The present results elucidate anticancer mechanism of PS-A and suggest that PS-A is a potential therapeutic or adjuvant strategy for the treatment of RCC patients.

## Figures and Tables

**Table 1:** Values of IC_50  growth_ and IC_50  migration_ of Chinese herb extracts at 24 h for 786O cells.

	IC_50 growth_ (mg/mL)	IC_50 migration_ (mg/mL)	IC_50 growth_/IC_50 migration_
*Paeonia suffruticosa*	1.5	0.3	5.0
*Trichosanthes kirilowii*	30.0	6.2	4.8
*San jhong kuei jian tang*	22.4	9.8	2.3
*Taraxacum monogolicum*	3.8	2.3	1.7
*Kalimeris indica*	4.8	3.7	1.3
*Platycodi radix*	2.1	1.7	1.2
*Abrus precatorius*	5.9	—	—
*Catharanthus roseus *	25.1	—	—
*Andrographis paniculata*	—	—	—
*Curcuma zedoaria*	—	—	—
